# The Aging Performance of PVDF in Acid Oil and Gas Medium

**DOI:** 10.3390/polym14194244

**Published:** 2022-10-10

**Authors:** Guoquan Qi, Hongxia Yan, Dongtao Qi, Houbu Li, Zhao Zhang

**Affiliations:** 1 State Key Laboratory of Performance and Structural Safety for Petroleum Tubular Goods and Equipment Materials, CNPC Tubular Goods Research Institute, Xi’an 710077, China; 2 Department of Applied Chemistry, School of Chemistry and Chemical Engineering, Northwestern Polytechnical University, Xi’an 710072, China

**Keywords:** PVDF, H_2_S, autoclave, exposure test, yield stress, macromechanical performance, thermal performance

## Abstract

In the process of transporting oil and gas, the service performance of thermoplastic pipes will decline due to the multiple influences of medium, temperature, and pressure. In order to study the service performance changes of PVDF pipes under oil and gas transportation conditions, the high-temperature autoclave is used to simulate the service state of the pipe in the mediums. The PVDF samples are exposed to simulated oil and gas mediums for 1 week, 3 weeks, 5 weeks, and 7 weeks under 60 °C and 90 °C conditions. After the exposure test, the physical and chemical properties of the PVDF pipe are tested and compared with the initial samples. Compared with the initial sample, the sample quality after the exposure test has a slight increase, with growth rates of 2% and 3% at 60 °C and 90 °C, respectively. Meanwhile, the tensile strength of the samples is about 13% and 21% lower than that of the initial sample, respectively. According to the microscopic morphology analyses, there are some crack defects on the surface of the sample after the exposure test. Through infrared analysis, it is shown that no molecular chain breakage, crosslinking, or other reactions are found after the exposure test. The above analysis shows that the PVDF sample has slight penetration and swelling during the exposure test. However, due to the large force between the PVDF molecules, its mechanical properties have a small downward trend, showing excellent environmental stress crack resistance.

## 1. Introduction

In recent years, with the increasing depth of oil and gas exploration, the temperature of the oil and gas medium has also increased, and due to a large number of acid medium comprehensive effects, the corrosion of metal pipelines has become more intensified [1]. Due to the excellent corrosion resistance of thermoplastics and their reinforced composite pipes, they have been widely used in the field of gathering and transportation of oil and gas fields, and they have become one of the important solutions to the corrosion problem in recent years. However, because of the poor temperature resistance and aging in long-term service, failure events of thermoplastic materials occur from time to time [2,3,4,5,6]. Therefore, high-performance plastics, such as PVDF, have entered the scope of people’s choice of materials to replace the thermoplastics with lower-temperature resistance. The chemical structure of PVDF with C-F bonds can form the most stable and firm bond with H^+^ [7,8,9,10]. Therefore, PVDF has special physical and chemical properties, not only having strong wear resistance and impact resistance, but also high aging resistance in extremely harsh environments.

However, PVDF has been used in acid oil and gas environments for a short period of time and lacks relevant data support [11]. In order to increase the rationalization and large-scale application of thermoplastics and their composite pipes, the aging behavior of PVDF with excellent temperature resistance and medium permeability resistance was studied, and its change rule was revealed.

In order to simulate the state of PVDF pipes under service conditions, high-temperature autoclave equipment was used in exposure tests. During the test, the real working environment was simulated by adjusting various parameters such as temperature, pressure, and medium. Through the exposure test, the changes in various properties of the sample in the multiphysical field environment (temperature, pressure, and medium) can be analyzed, and the attenuation law of PVDF pipe properties under the comprehensive action of temperature, pressure, and medium can be clarified. Further, the relationship between microstructure and mechanical properties can be analyzed, and the results can be used to guide the development of new products and the scientific selection of materials.

## 2. Experimental

### 2.1. Sample Preparation

**Figure 1:**
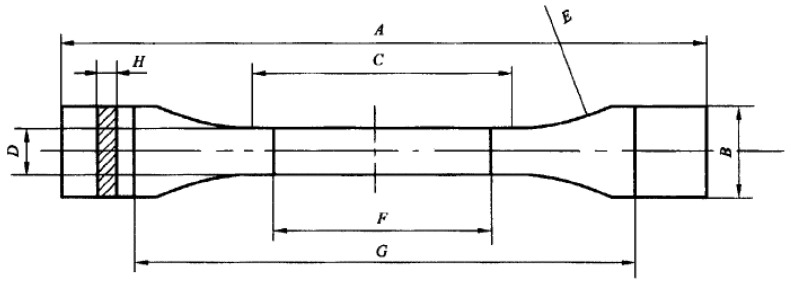
Sample size.

In this study, the pipe was PVDF with the specification of DN100*6 mm produced by Solvay Co. Dumbbell-shaped samples were cut from PVDF pipes, and the preparation requirements were in accordance with ISO 6259-3:1997 “Thermoplastics pipes—Determination of tensile properties—Part 3: Polyolefin pipes”. The sample type and size requirements are shown in Figure 1 and Table 1. There were four samples under each test condition, including three for the mechanical property test and one for structural and composition analysis.

### 2.2. Test Process

The exposure test was carried out by the high-temperature autoclave equipment under the simulated medium environment of acid oil and gas fields, which included three parts: performance test before aging test, aging test, and performance test after aging test. It is described in detail as follows:

Performance test before aging test: The test items included samples size, weight, composition, microstructure, tensile properties, and thermal stability.

Aging test: The exposure test was completed with the autoclave equipment, and the medium selected refers to ISO 23936-1:2009 “Petroleum, petrochemical and natural gas industries Non-metallic materials in contact with media related to oil and gas production Part 1: Thermoplastics”. The requirements for the test medium are shown in Table 2. In this study, the pressure was 8 MPa, while the temperature was 60 °C and 90 °C. Under the two temperature conditions, the test time was 1 week, 3 weeks, 5 weeks, and 7 weeks.

Performance test after aging test: The test items were the same as those before the test.

### 2.3. Performance Test and Characterization

#### 2.3.1. Weight Change

The weight of the sample before and after the exposure test was tested by the electronic analytical balance (CPA225D, Sartorius, Gottingen, Germany). The accuracy of the electronic analytical balance was 10^−4^ g. After the test was completed, the weight change of different exposure times was compared and analyzed.

#### 2.3.2. Macromechanical Performance

The macromechanical performance was tested by the universal test machine (UH-F500KNI, Xinsansi Co., Ltd., Shanghai, China) with the accuracy of 1N according to ASTM 638. The test data included strain and stress. The same batch of mechanical tensile tests was performed under the tensile rate of 50 mm/min.

#### 2.3.3. Thermal Performance Analysis

The thermal performance of the sample after the exposure test was tested by the Vicat softening point tester (Precision Co., Ltd., Chengde, China) and the differential scanning calorimeter (DSC, TA Q200, New Castle, DE, USA). For the Vicat softening temperature test, refer to the standard ISO 306. Among the test parameters, the force was 50 N, the heating rate was 50 °C/h, and the test temperature range was 25 °C to 200 °C. When the depth of the pressure needle penetrating the sample exceeds the initial position by 1 mm, the corresponding test temperature is the Vicat softening temperature.

In order to record and test the crystallization melting curve of PVDF pipe, the thermal scanning curve was analyzed. In the DSC analysis, the heating and cooling curves were scanned at a rate of 10 °C/min under the protection of N_2_, and the test temperature range was 25 °C to 240 °C. The change in crystallinity was analyzed by measuring the change in enthalpy of fusion of the polymer. The calculation formula is as follows:

$$x_{c}=\frac{\Delta H}{\Delta H_{0}}\times 100\%$$

where ∆*H* and ∆*H*_0_ are the enthalpy of fusion of the PVDF samples and 100% crystalline samples, respectively.

#### 2.3.4. Composition Analysis

The composition was tested by Fourier transform infrared spectroscopy (FT-IR, Avator 360, Thermo Nicolet Co., Ltd., Madison, WI, USA). The spectral range of the FT-IR was 400 cm^−1^ to 4000 cm^−1^ and the spectral resolution was 4 cm^−1^.

#### 2.3.5. Microstructure Characterization

The microstructure was characterized by a scanning electron microscope (SEM, S4800, Hitachi Co., Ltd., Tokyo, Japan). In order to avoid the influence of the media attached to the surface on the SEM observation, ultrasonic cleaning was carried out before the observation.

## 3. Results and Discussion

### 3.1. Weight Change

**Figure 2:**
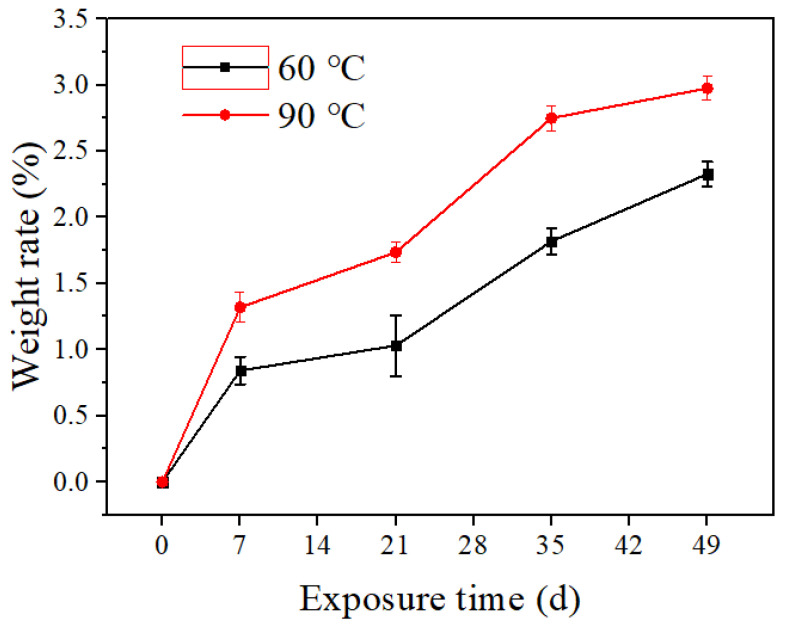
The weight change of PVDF after the exposure test.

The test result of the weight change rate is shown in Figure 2. As the exposure time increases, the rate of weight change increases. When the exposure time is 7 weeks, the rate of weight change reaches the maximum value with an average value of 3%, but it still meets the requirements of the standard ISO 23936.1 (±5%). As the exposure time increases, the reason for the increase in weight change rate is the penetration of the medium into the PVDF pipe. During the exposure test, the weight increment and loss occurs simultaneously. The weight increment is caused by the penetration of the medium into the interior. The weight loss is caused by the small uncured or decomposed molecules inside the polymer, which are easily extracted by the medium. As the swelling phenomenon of polymer materials during exposure is greater than the effect of small molecules being extracted, the quality of the sample increases slightly [12,13]. With the continuous increase in exposure time, the increase in weight change rate shows a decreasing trend, because the medium entering the sample tends to be saturated.

### 3.2. Macromechanical Performance

**Figure 3:**
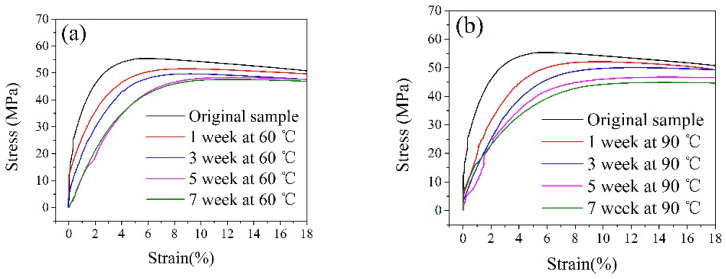
The stress–strain curves of PVDF samples at different exposure times: (**a**) 60 °C; (**b**) 90 °C.

**Figure 4:**
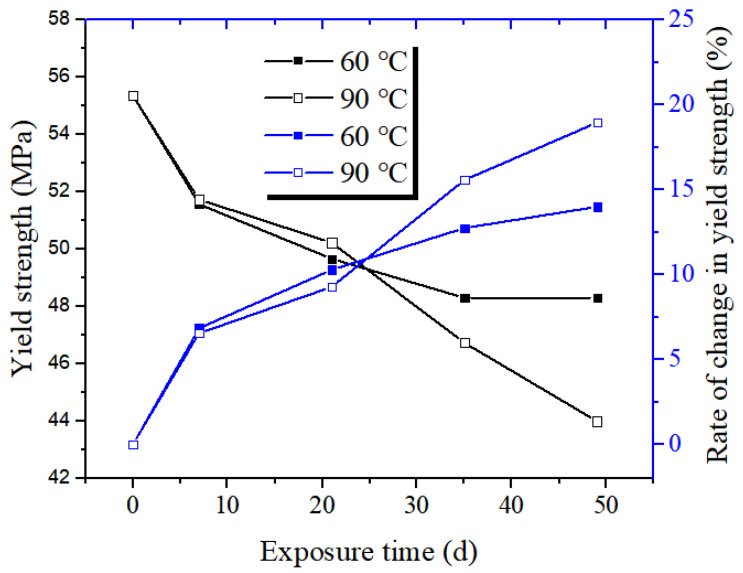
Yield strength and its change rate of PVDF samples at different exposure times.

The stress–strain curves of PVDF pipe at different exposure times are shown in Figure 3. With the increase in test time (0 to 7 weeks), under the same strain value, the tensile strength value of the PVDF pipe shows a downward trend, but its change rate is greater in the early period (0 weeks to 1 week), while it is more moderate in the later period (3–7 weeks), as shown in Figure 4. The corresponding yield strength (48.31 MPa) decreases by about 13% compared with the initial sample (55.40 MPa) when the test time is 7 weeks at the test temperature of 60 °C. In comparison, at 90 °C, the yield strength (43.99 MPa) corresponding to the exposure time of 7 weeks decreases by about 21% compared with the initial sample (55.40 MPa). Under general conditions, the breakage of polymer materials is mainly due to chemical bond destruction, inter-molecular slippage, and the van der Waals force or hydrogen bond destruction. During the exposure test, due to the effects of medium penetration and swelling, various defects such as microholes are formed inside of the pipe to cause a concentration effect [14,15,16]. The stress on the molecular chain near the hole exceeds the average stress of the actual material by dozens of times, much higher than the average stress. The pipe would break down first in these areas and then extend to the entire pipe. Consistent with the behavior of other thermoplastics, such as PE and PA, the increase in temperature also promotes the decrease in mechanical properties [17,18]. However, under the conditions of 60 °C and 90 °C, the yield strength of the PVDF pipe sample is still high after the exposure test, as shown in Figure 4. It can be seen that its mechanical properties and stability have obvious advantages.

### 3.3. Thermal Performance Analysis

#### 3.3.1. Vicat Softening Temperature

**Figure 5:**
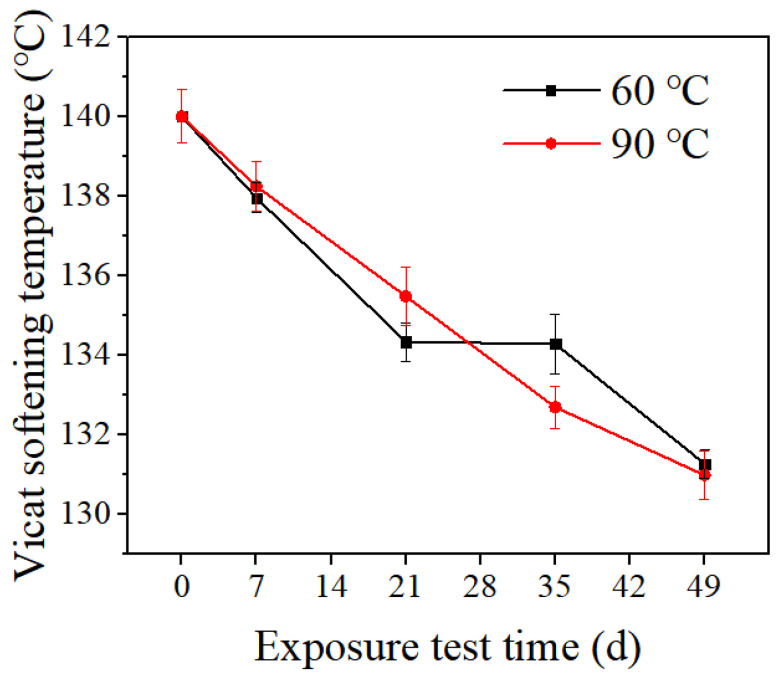
Vicat temperature of PVDF samples after the exposure test.

**Figure 6:**
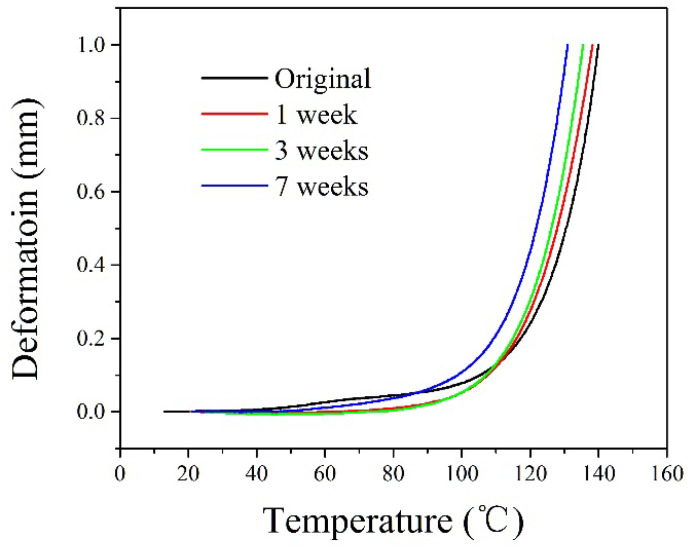
The Vicat softening temperature test results.

The Vicat softening temperature test results are shown in Figure 5. As the exposure time increases, the Vicat softening temperature decreases continually. The Vicat softening temperatures of the sample after exposing for 7 weeks are about 6.2% and 6.4% lower than those of the initial sample at 60 °C and 90 °C, respectively. In Figure 6, the Vicat softening temperature curve of all the samples shows three stages. The initial stage is between 20 °C and 100 °C, the second stage is between 100 °C and 120 °C, and the third stage is between 120 °C and the Vicat softening temperature. In the first stage, with the increase in heating temperature, the indentation depth does not increase significantly, and the temperature basically has no effect on the dimensional stability of the material. The second stage is a slowly rising deformation, and the displacement is more obvious in this stage, indicating that its dimensional stability becomes worse as the temperature increases. In the third stage, the temperature is most obviously affected by the dimensional stability. Compared with the other two stages, this stage has the worst dimensional stability. In practical applications, it should be avoided under this temperature condition. The Vicat softening temperature is one of the important indexes to evaluate the heat resistance of materials and reflects the mechanical properties of polymers. The higher the Vicat softening temperature is, the better the dimensional stability of the material when heated, and the smaller the thermal deformation is. Due to the swelling effect of the medium during the exposure test, the dimensional stability of the material deteriorates under heating conditions [19,20].

#### 3.3.2. Melting and Crystallization Curve

**Figure 7:**
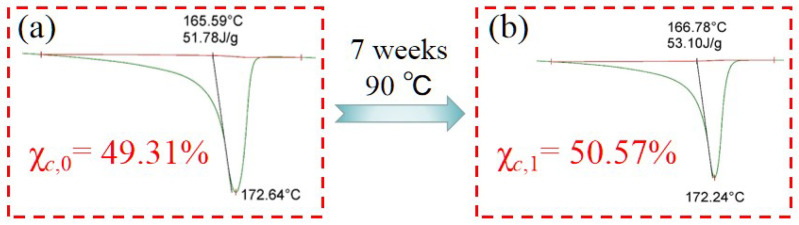
Melting curve of PVDF samples: (**a**) the original sample; (**b**) the sample exposed for 7 weeks at 90 °C.

To check the possible alteration in the crystallinity due to the exposure test, DSC was been performed to check the crystallinity of the two samples (the original sample and the sample exposed for 7 weeks) in comparison (shown in Figure 7). The thermograph of the sample after exposing for 7 weeks shows thermal values (melting peaks, crystallization peak, and enthalpies of fusion and crystallization) that are comparable to the original sample. The crystallinity of the polymer is proportional to its enthalpies of fusion, so the crystallinity values of the two samples are basically the same. It shows that the exposure test has little effect on the order of the internal structure of the polymer [21,22,23].

### 3.4. Composition Analysis

**Figure 8:**
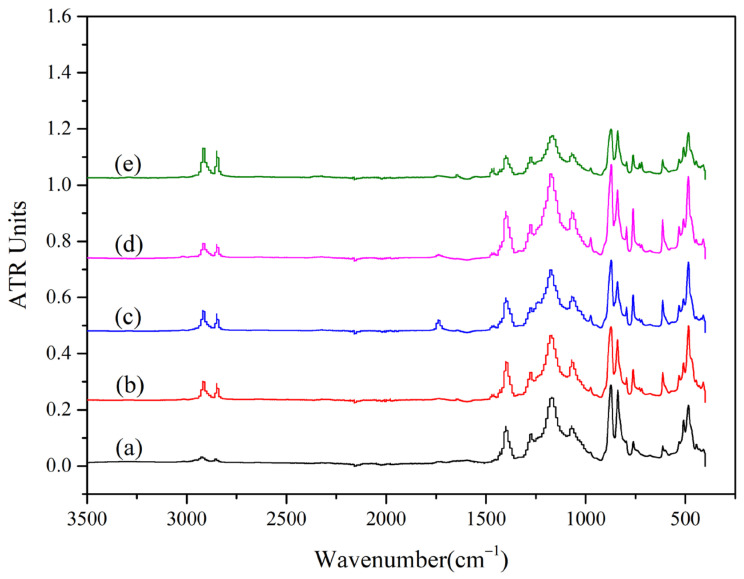
The FT-IR spectra of PVDF: (a–e) exposure time of 0, 1, 3, 5, and 7 weeks.

In order to analyze the changes in the composition of PVDF pipes after the exposure test, the functional groups of the pipes were characterized by FT-IR spectroscopy, and the results are shown in Figure 8. The FT-IR spectra contain five samples with exposure times of 0, 1, 3, 5, and 7 weeks. Compared with the results of all samples, the infrared spectra of the five samples are basically the same. After the PVDF is exposed at different times, the peak position and intensity of the respective characteristic areas and fingerprint areas do not change significantly, indicating that the internal structure of PVDF is still stable with the increase in exposure time under the simulated oil field environment.

### 3.5. Microstructure Characterization

**Figure 9:**
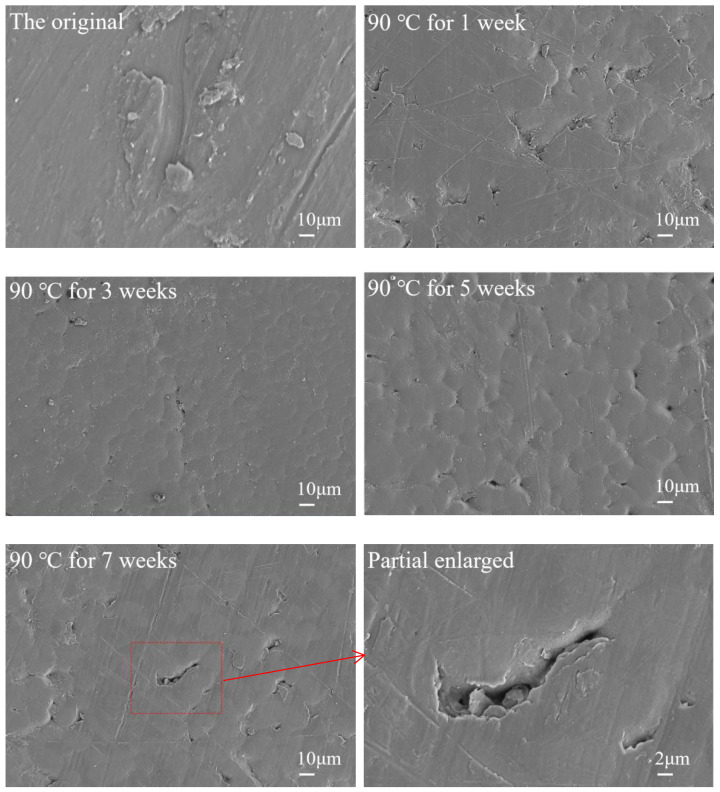
The SEM micrographs of PVDF.

Figure 8 shows SEM observations of the samples before and after the exposure test. It can be seen from Figure 9 that the pitting phenomenon appears on the surface of the samples under different exposure times, while the original sample does not have any pitting. The phenomenon, caused by the extraction of the material in the amorphous region by the medium, becomes more obvious as the exposure time increases. PVDF polymer is composed of a crystalline and amorphous part. In the crystalline phase, macromolecules are arranged closely and orderly, which is strong and inactive. In the amorphous phase, it is very easy to erode by the medium first, due to its structural discontinuity. The phenomenon of micropits between the grain boundaries appears, and finally, a penetrating channel is formed, which leads to the swelling phenomenon of the whole sample [24,25,26,27]. The additives and other substances in the amorphous area are replaced to the outside of the sample, thereby forming microholes in the amorphous area.

### 3.6. Comprehensive Analysis

Through the comparison of the PVDF physical properties, the composition, and the microscopic morphology before and after the exposure test, the performance change trend was analyzed with increasing exposure time. Regardless of whether it is in a simulation test or in an actual service, the decline in pipe performance is mainly affected by the three factors, which are the medium type, temperature, and pressure [27]. During long-term exposure, the medium will enter the inside of the pipe due to penetration [28]. From a microscopic point of view, the medium enters the grain boundary area of the pipe inside, resulting in swelling of the material, which is presented as a weight increase. At the same time, it results in extraction and decomposition of the small uncured resin molecules, which is presented as a weight loss. The swelling effect is obviously stronger than the extraction and decomposition effect, and the final result is that the mass increases with the increase in exposure time.

Due to the effect of medium penetration and swelling, various defects such as microholes are formed inside the PVDF to cause a concentration effect. The stress of the molecular chain is concentrated at the defects position, and the stress that the molecular chain can withstand is higher than at other locations. Fracture failure occurs first in the stress concentration area, which ultimately leads to a decrease in the overall mechanical properties. As the swelling process is only a physical process and no chemical reaction occurs, it can also be proven from the infrared analysis. The analysis of micromorphology confirmed the existence of microcrack defects.

**Figure 10:**
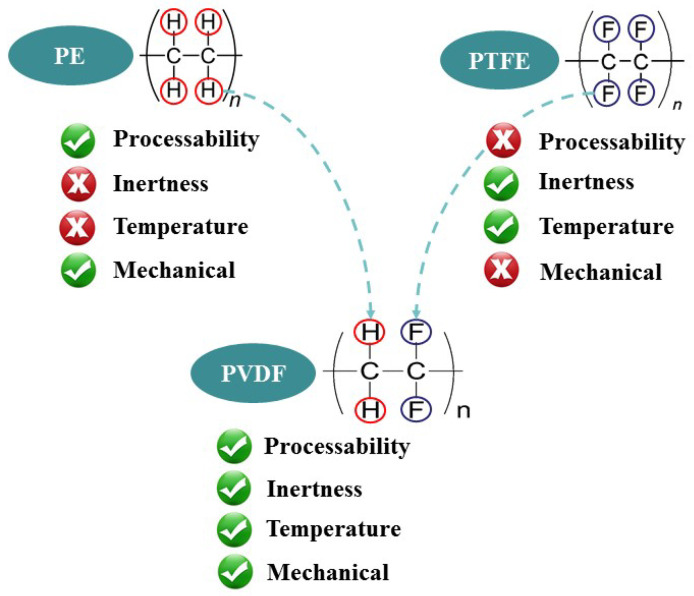
PVDF performance diagram.

However, as the microcracks are microsized and morphological, they have little effect on reducing the macromechanical properties. In actual service, the microcracks will not cause service performance obviously. The reason is mainly due to its structure. The C-F bond and C-H bond in the PVDF structure are arranged in an interactive manner, with certain structural regularity. It has the excellent properties of PE and PTFE, and has good flexibility, intermolecular force, temperature resistance, and mechanical properties, as shown in Figure 10. In particular, the PVDF monomer contains two C-F bonds (its bond energy is 486 kJ/mol), which are covalently bonded, and its long-term service stability is greatly improved compared with polyolefin pipes composed of C-H bonds. In addition, when the PVDF molecular chains are arranged, the distance between the segments is close, and there are a large number of hydrogen bonds with high bond energy. Therefore, PVDF molecules have a large force, and its cohesive energy density is about 538.24 MJ/m^3^. Due to the strong force between molecules, PVDF has excellent temperature resistance, mechanical properties, and corrosion resistance.

## 4. Conclusions

After exposure tests at two temperatures (60 °C and 90 °C) and different exposure cycles (1 week, 3 weeks, 5 weeks, and 7 weeks), the service performance of test samples decreased slightly with the increase in exposure time. When exposed at 60 °C and 90 °C for 7 weeks, the average change rate of mass was 2.3% and 3.0%, respectively. The yield strength decreased by 13% and 21%, respectively. The decrease rates of the Vicat softening temperature were 6.2% and 6.4%, respectively. Due to the strong force between PVDF molecules and the relatively regular condensed structure, PVDF showed an excellent environmental stress cracking resistance ability.

## Figures and Tables

**Table 1:** Sample size description.

Serial Number	Description	Size (mm)
A	Minimum total length	150
B	End width	20 ± 0.2
C	Parallel part length	60 ± 0.5
D	Parallel part width	10 ± 0.2
E	Radius	60
F	Distance between markings	50 ± 0.5
G	Distance between clamps	115 ± 0.5
H	Wall thickness	wall thickness of the pipe

**Table 2:** The requirements for the test medium.

Test Medium	Composition	Proportion (%)
Gas phase (30%)	CO_2_	10
	H_2_S	10
	CH_4_	80
Liquid phase (70%)	Heptane	70
	Cyclohexane	20
	Toluene	10

## Data Availability

The data presented in this study are available on request from the corrsponding author.
